# Supplementary material for: Diversity, distribution and dynamics of large trees across an old-growth lowland tropical rain forest landscape
Source: PLoS One. 2019 Nov 11;14(11):e0224896. doi: 10.1371/journal.pone.0224896 (PMC6844552; doi:10.1371/journal.pone.0224896)
Supplement: S5 Table — (DOCX) [file pone.0224896.s005.docx]

S5 Table. Large tree (diameter >60 cm) density by soil type. Data are the mean number of large trees per 0.50 ha large tree inventory plot, and their summed basal area (N=238 plots).

|  |  |  |  |
| --- | --- | --- | --- |
| **Soil_type** | **N plots** | **Mean number large trees** | **Mean plot large tree basal area (m^2^)** |
| Old Alluvial | 30 | 9.3 | 4.4 |
| Residual Soils | 205 | 6.4 | 2.9 |
| Stream-associated | 3 | 11.3 | 4.9 |
