# Supplementary material for: Diversity, distribution and dynamics of large trees across an old-growth lowland tropical rain forest landscape
Source: PLoS One. 2019 Nov 11;14(11):e0224896. doi: 10.1371/journal.pone.0224896 (PMC6844552; doi:10.1371/journal.pone.0224896)
Supplement: S6 Table — (DOCX) [file pone.0224896.s006.docx]

S6 Table. Total basal area loss by size class.

| Year | N Dead | BA loss  smaller stems | BA loss  large trees | Total  BA loss | % of loss  By large trees |
| --- | --- | --- | --- | --- | --- |
| 1998 | 171 | 7.30 | 1.78 | 9.08 | 19.60 |
| 1999 | 141 | 4.73 | 0.42 | 5.15 | 8.16 |
| 2000 | 106 | 3.01 | 3.84 | 6.85 | 56.06 |
| 2001 | 88 | 3.62 | 0.31 | 3.93 | 7.89 |
| 2002 | 112 | 3.84 | 1.15 | 4.99 | 23.05 |
| 2003 | 95 | 3.34 | 1.10 | 4.44 | 24.77 |
| 2004 | 152 | 5.89 | 2.44 | 8.33 | 29.29 |
| 2005 | 104 | 4.38 | 0.40 | 4.78 | 8.37 |
| 2006 | 90 | 3.09 | 0.85 | 3.94 | 21.57 |
| 2007 | 96 | 3.86 | 2.29 | 6.15 | 37.24 |
| 2008 | 114 | 4.10 | 0.43 | 4.53 | 9.49 |
| 2009 | 92 | 3.02 | 0.36 | 3.38 | 10.65 |
| 2010 | 64 | 2.13 | 0.30 | 2.43 | 12.35 |
| 2011 | 88 | 2.61 | 0.64 | 3.25 | 19.69 |
| 2012 | 93 | 3.22 | 0.62 | 3.84 | 16.15 |
| 2013 | 81 | 2.94 | 2.07 | 5.01 | 41.32 |
| 2014 | 86 | 4.58 | 1.09 | 5.67 | 19.22 |
| 2015 | 127 | 5.31 | 0.91 | 6.22 | 14.63 |
| 2016 | 95 | 4.05 | 0.64 | 4.69 | 13.65 |
| 2017 | 100 | 3.65 | 2.14 | 5.79 | 36.96 |

Total basal area (BA) loss (m^2^) by large trees (diameter>60 cm) and smaller stems over an upland old-growth tropical rain forest landscape at the La Selva Biological Station, Costa Rica, 1997-2017. Data come from annual censuses of 18 0.5 ha plots (see text for site description).
