# Supplementary material for: Diversity, distribution and dynamics of large trees across an old-growth lowland tropical rain forest landscape
Source: PLoS One. 2019 Nov 11;14(11):e0224896. doi: 10.1371/journal.pone.0224896 (PMC6844552; doi:10.1371/journal.pone.0224896)
Supplement: S7 Table — (DOCX) [file pone.0224896.s007.docx]

S16 Table. Species-level annual mortality rates 2006-2016 for large trees in 238 0.50 ha plots in old-growth tropical rain forest at the La Selva Biological Station, Costa Rica. Individuals were first censused in 2006-2011, and re-censused in 2016 for survival. Species-level annual mortality rates were calculated for each year’s sample, and the weighted mean of these rates for each species was calculated (see text for statistical references).

| **Genus** | **Species** | **Family** | **Total sample of individuals** | **Weighted mean annual mortality** |
| --- | --- | --- | --- | --- |
| Pentaclethra | macroloba | Fabaceae | 831 | 2.47 |
| Balizia | elegans | Fabaceae | 74 | 1.87 |
| Carapa | nicaraguensis | Meliaceae | 63 | 2.56 |
| Virola | koschnyi | Myristicaceae | 45 | 1.51 |
| Vitex | cooperi | Lamiaceae | 43 | 2.27 |
| Laetia | procera | Salicaceae | 41 | 4.69 |
| Apeiba | membranacea | Malvaceae | 40 | 3.00 |
| Guarea | guidonia | Meliaceae | 39 | 0.18 |
| Stryphnodendron | microstachyum | Fabaceae | 32 | 9.59 |
| Vochysia | ferruginea | Vochysiaceae | 29 | 3.57 |
| Dipteryx | panamensis | Fabaceae | 29 | 0.04 |
| Inga | alba | Fabaceae | 26 | 5.65 |
| Ilex | skutchii | Aquifoliaceae | 24 | 0.52 |
| Hieronyma | alchorneoides | Phyllanthaceae | 24 | 1.20 |
| Lecythis | ampla | Lecythidaceae | 20 | 0.60 |
