# Supplementary figures and images for: Diversity, distribution and dynamics of large trees across an old-growth lowland tropical rain forest landscape
Source: PLoS One. 2019 Nov 11;14(11):e0224896. doi: 10.1371/journal.pone.0224896 (PMC6844552; doi:10.1371/journal.pone.0224896)

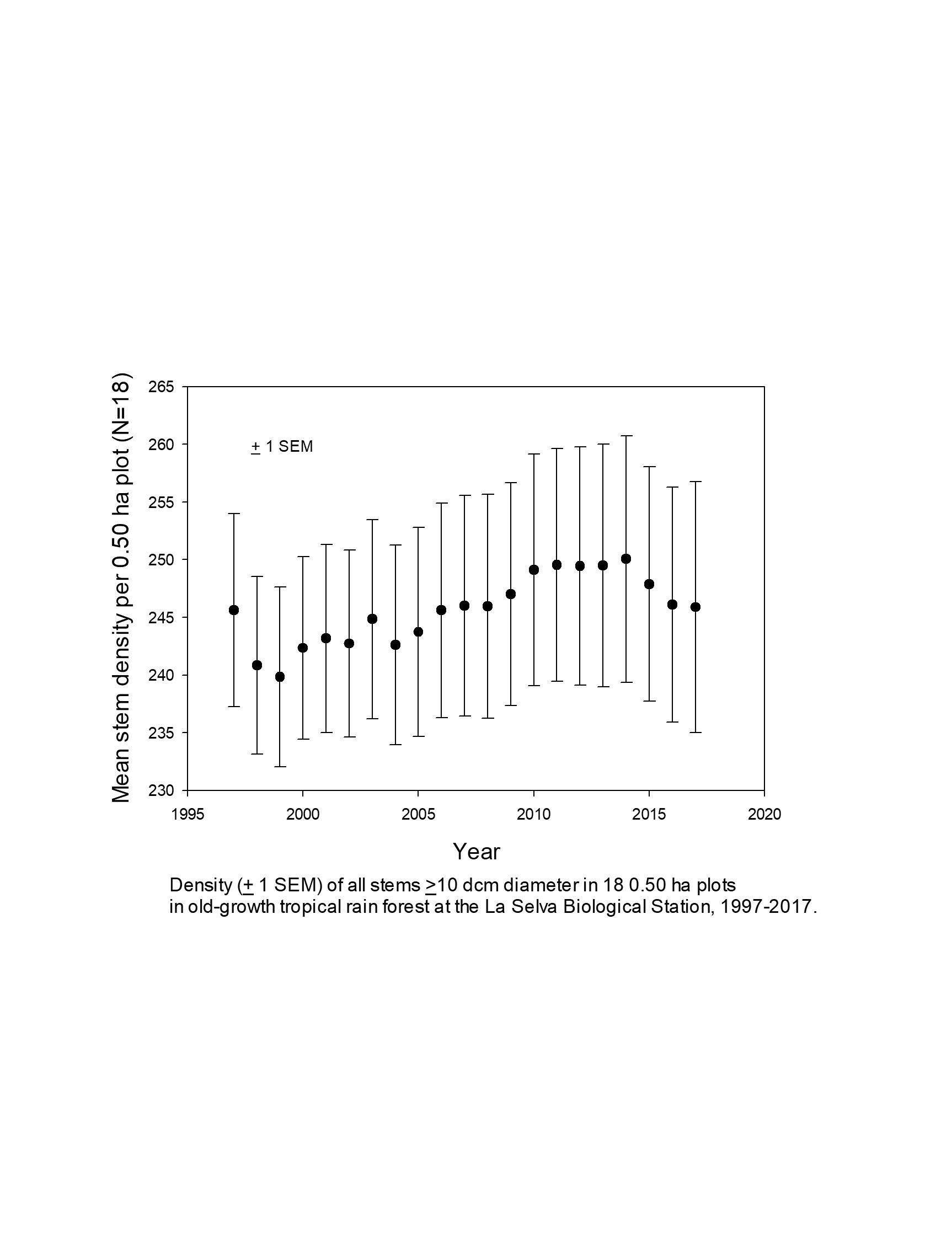

Supplement: S1 Fig — (TIF) [file pone.0224896.s008.tif]

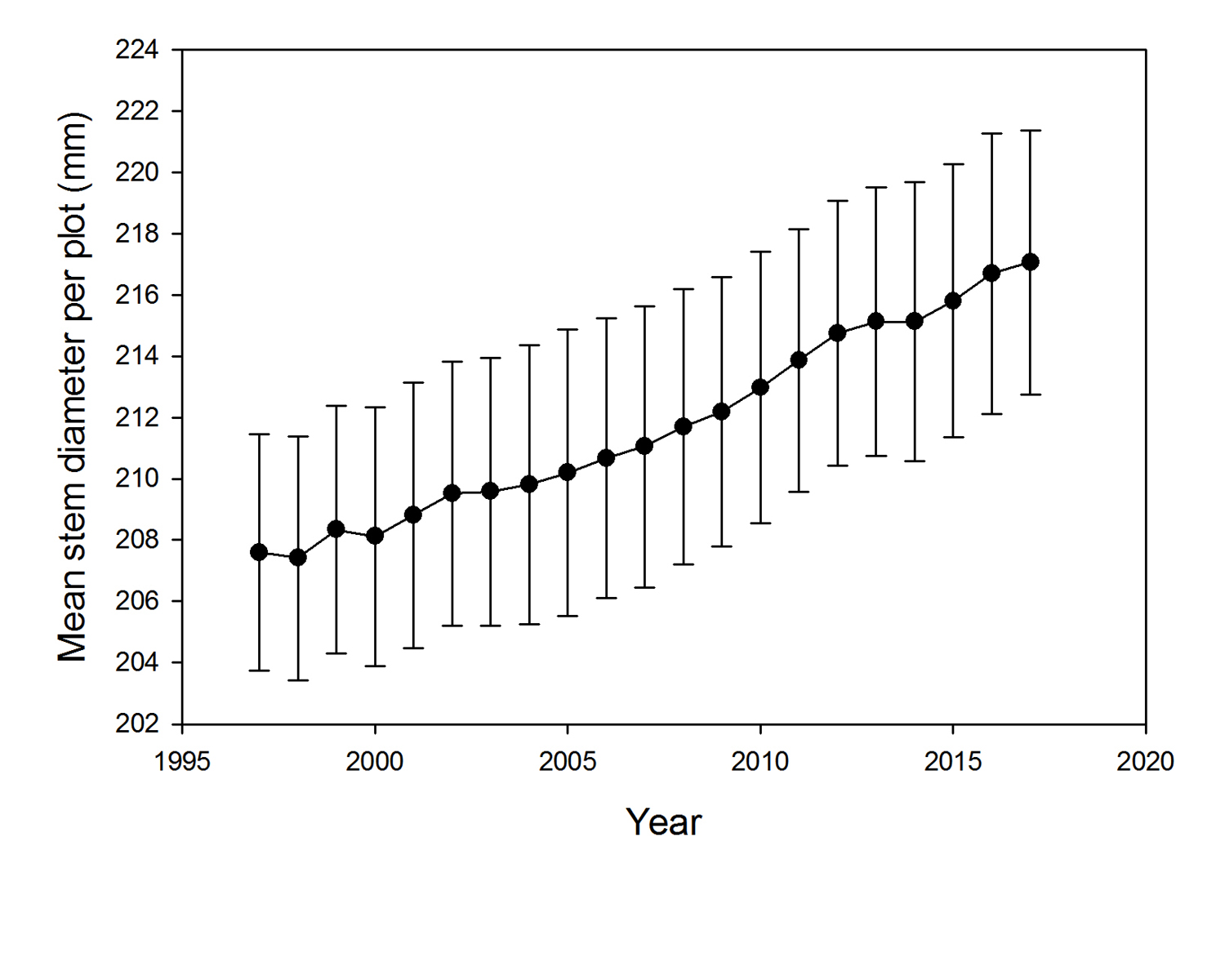

Supplement: S2 Fig — (TIF) [file pone.0224896.s009.tif]

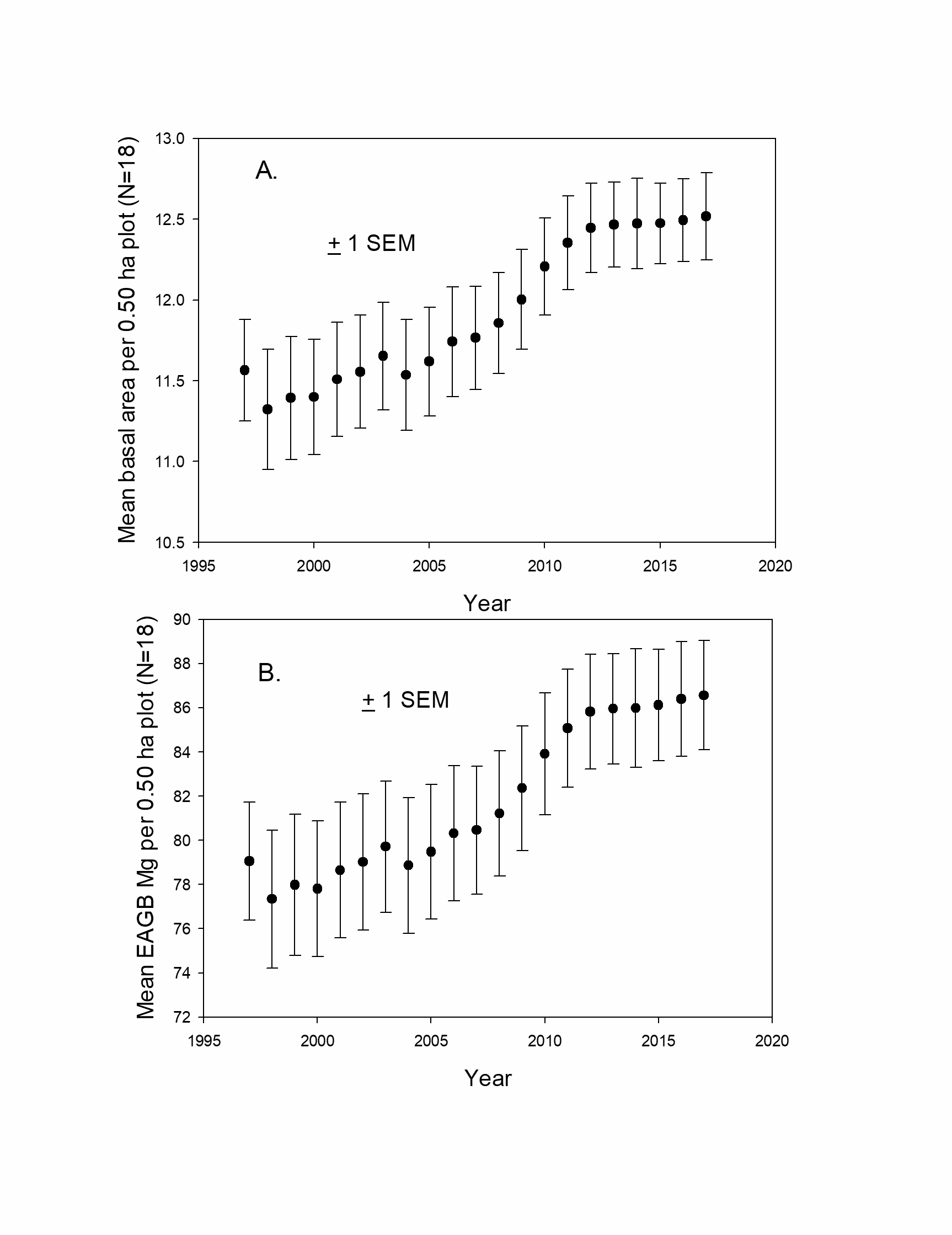

Supplement: S3 Fig — (TIF) [file pone.0224896.s010.tif]

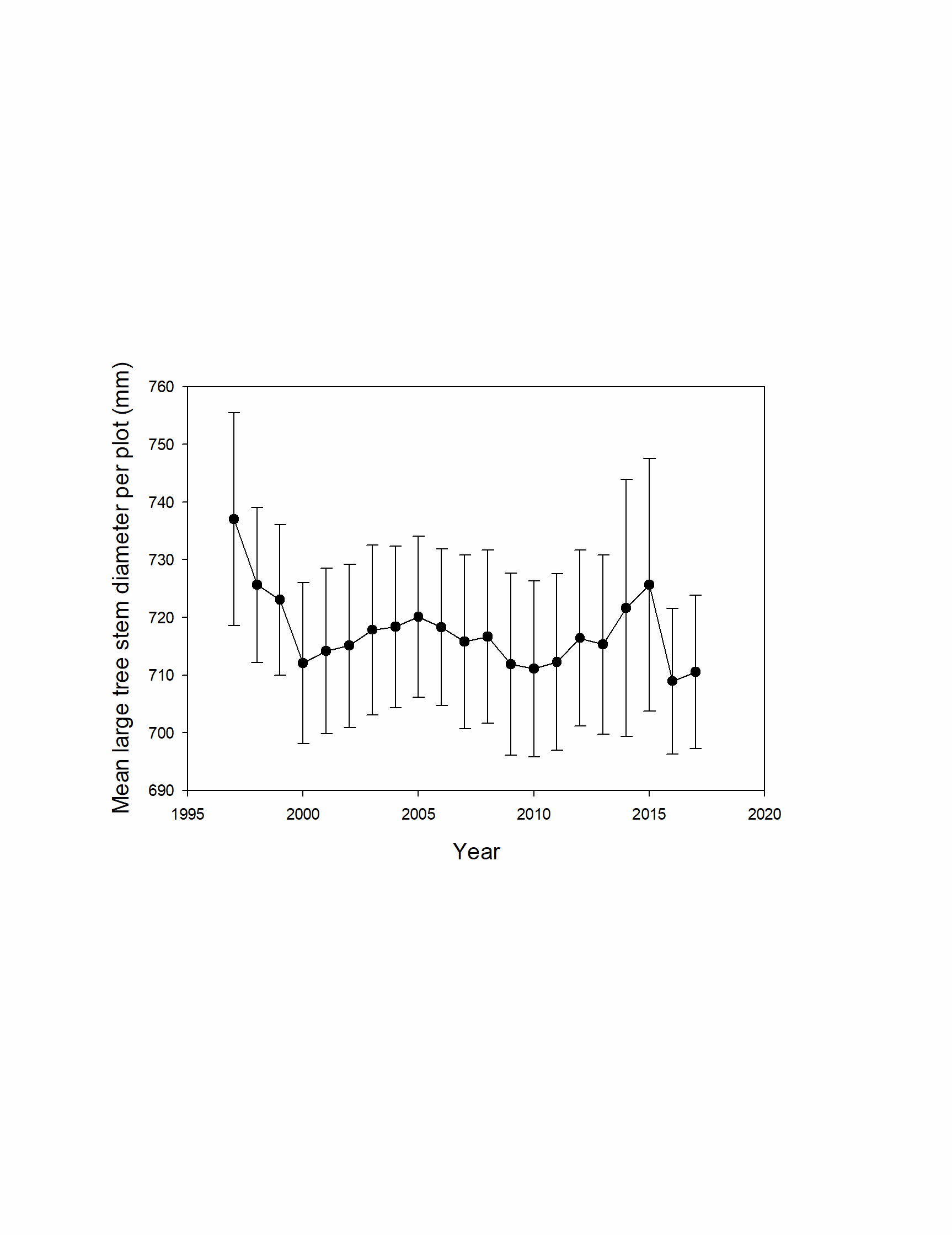

Supplement: S4 Fig — (TIF) [file pone.0224896.s011.tif]

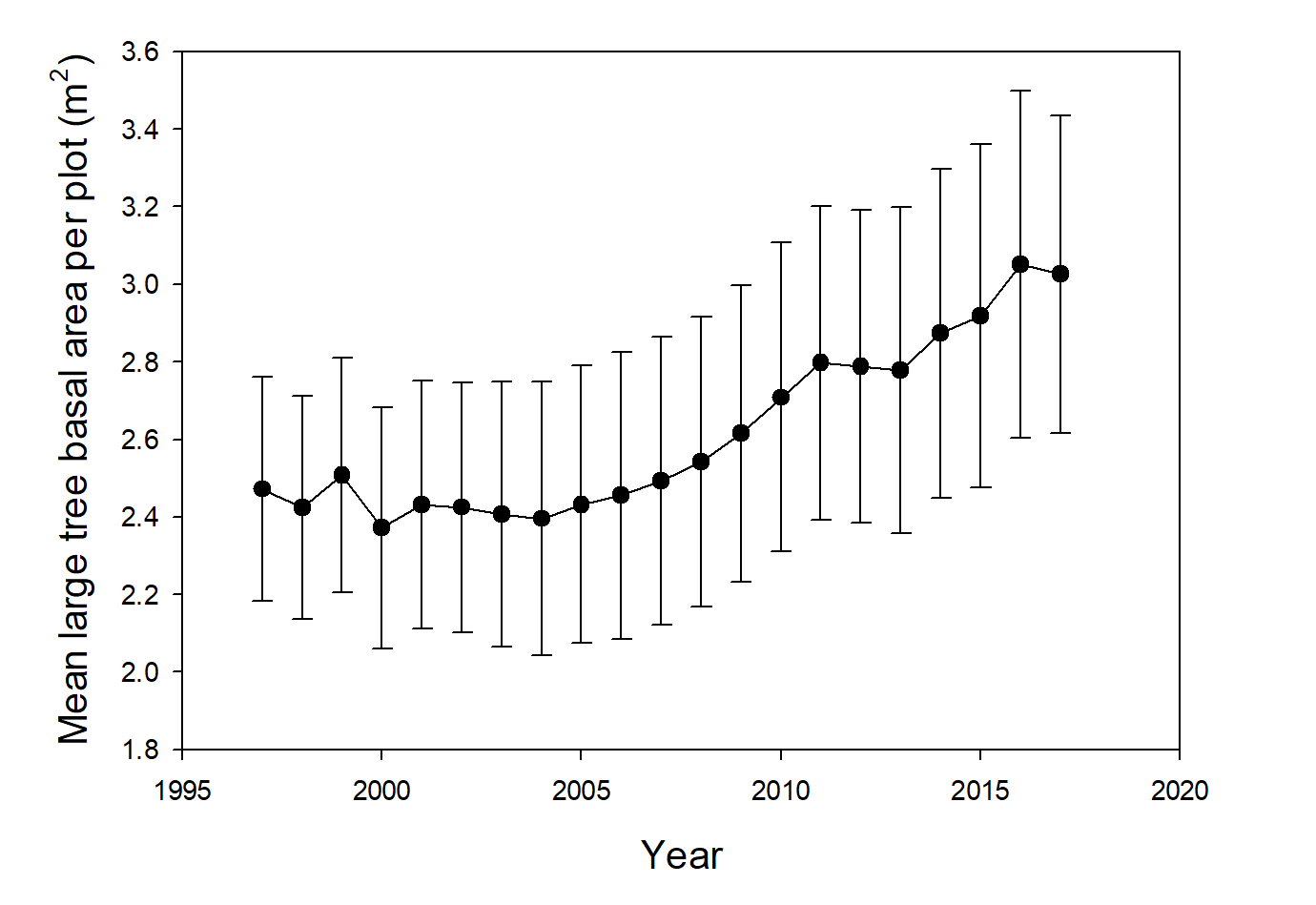

Supplement: S5 Fig — (TIF) [file pone.0224896.s012.tif]

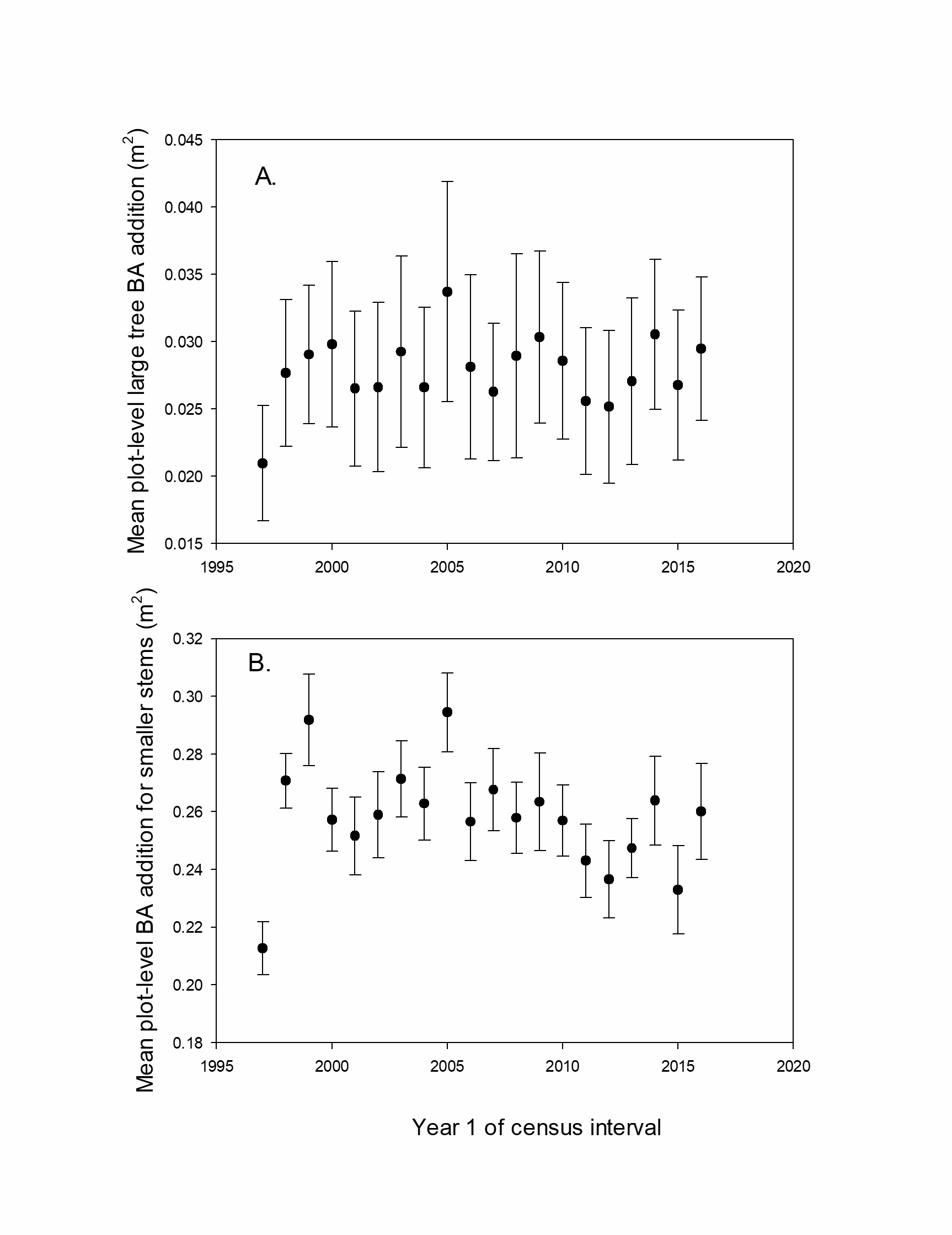

Supplement: S6 Fig — (TIF) [file pone.0224896.s013.tif]

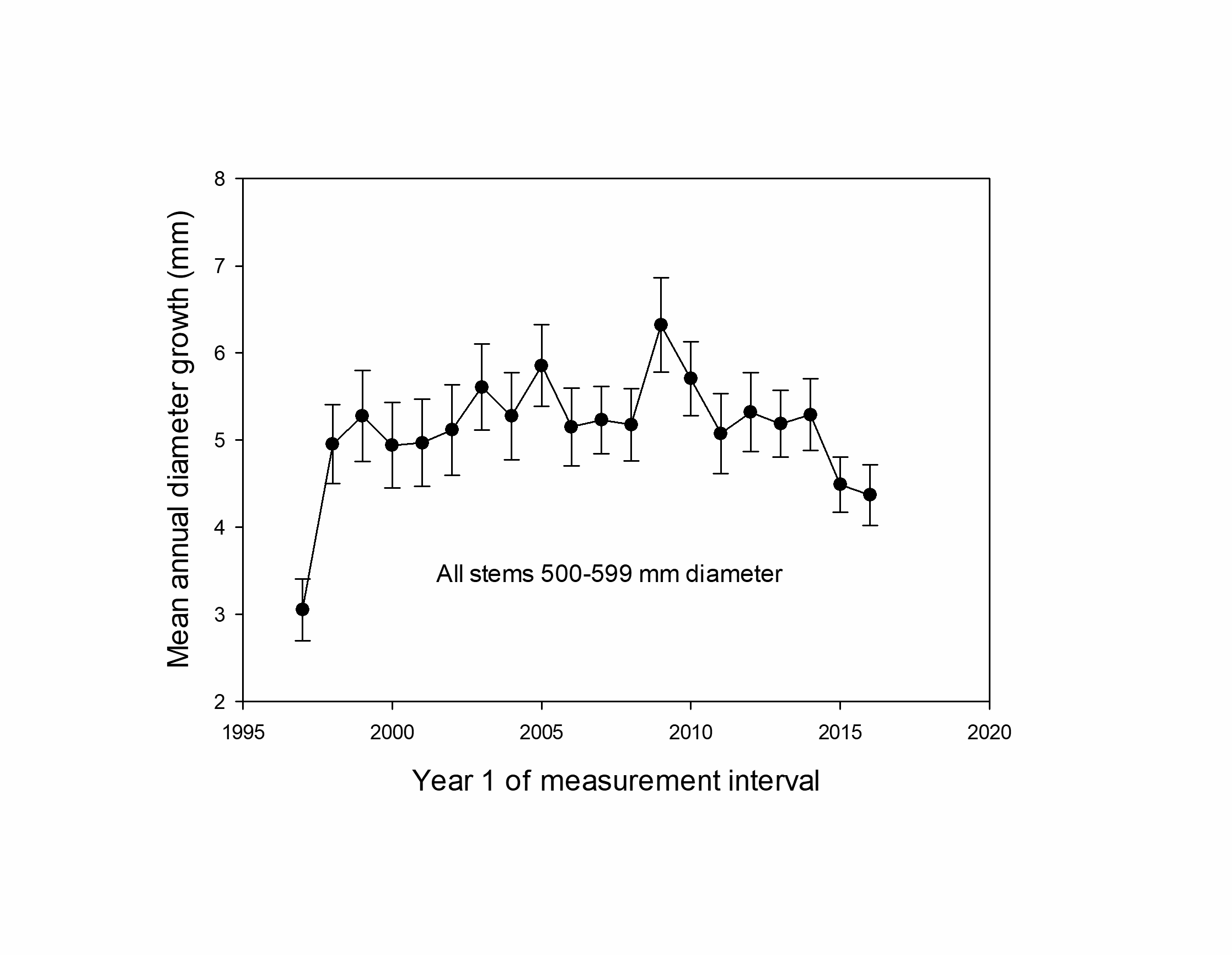

Supplement: S7 Fig — (TIF) [file pone.0224896.s014.tif]

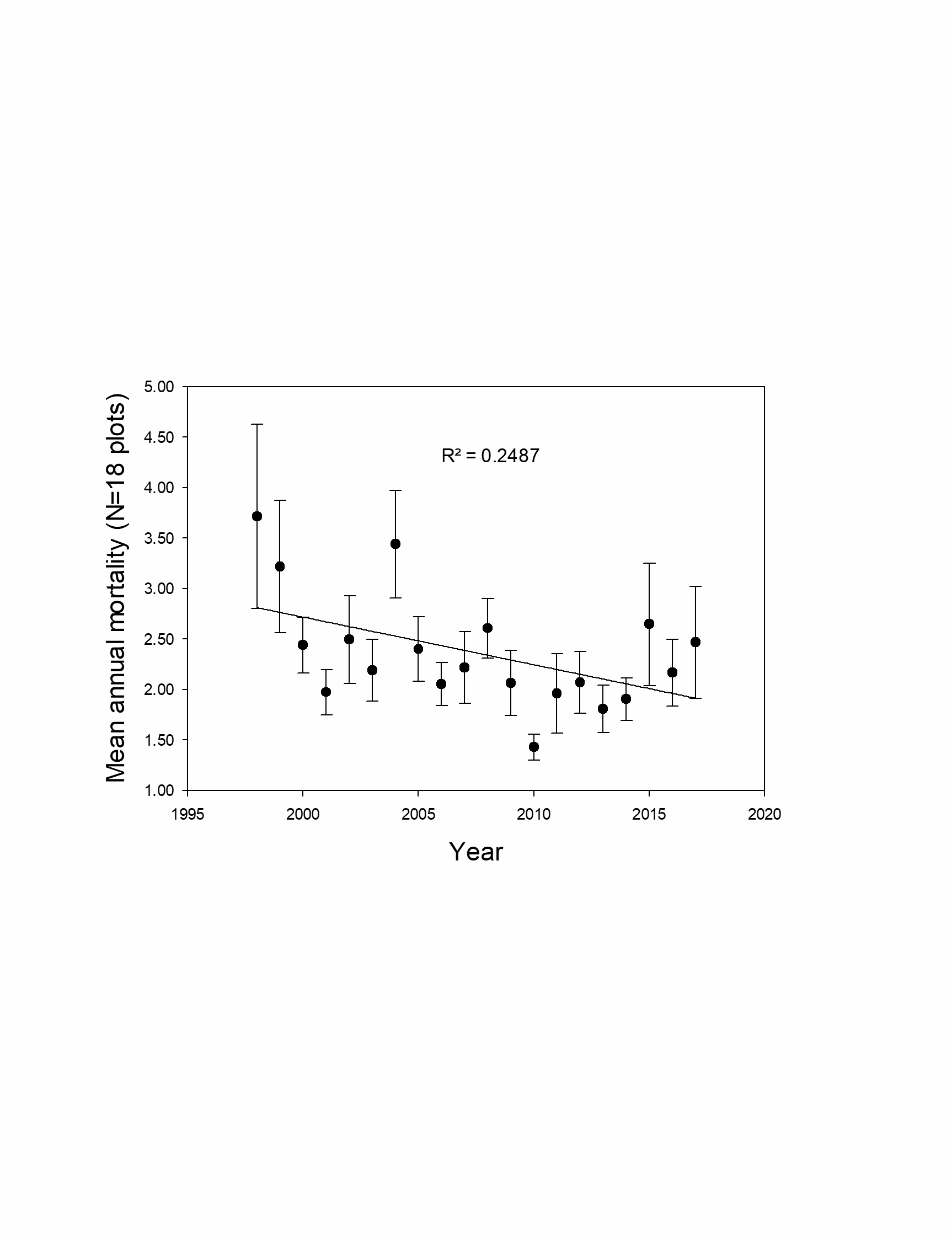

Supplement: S8 Fig — (TIF) [file pone.0224896.s015.tif]

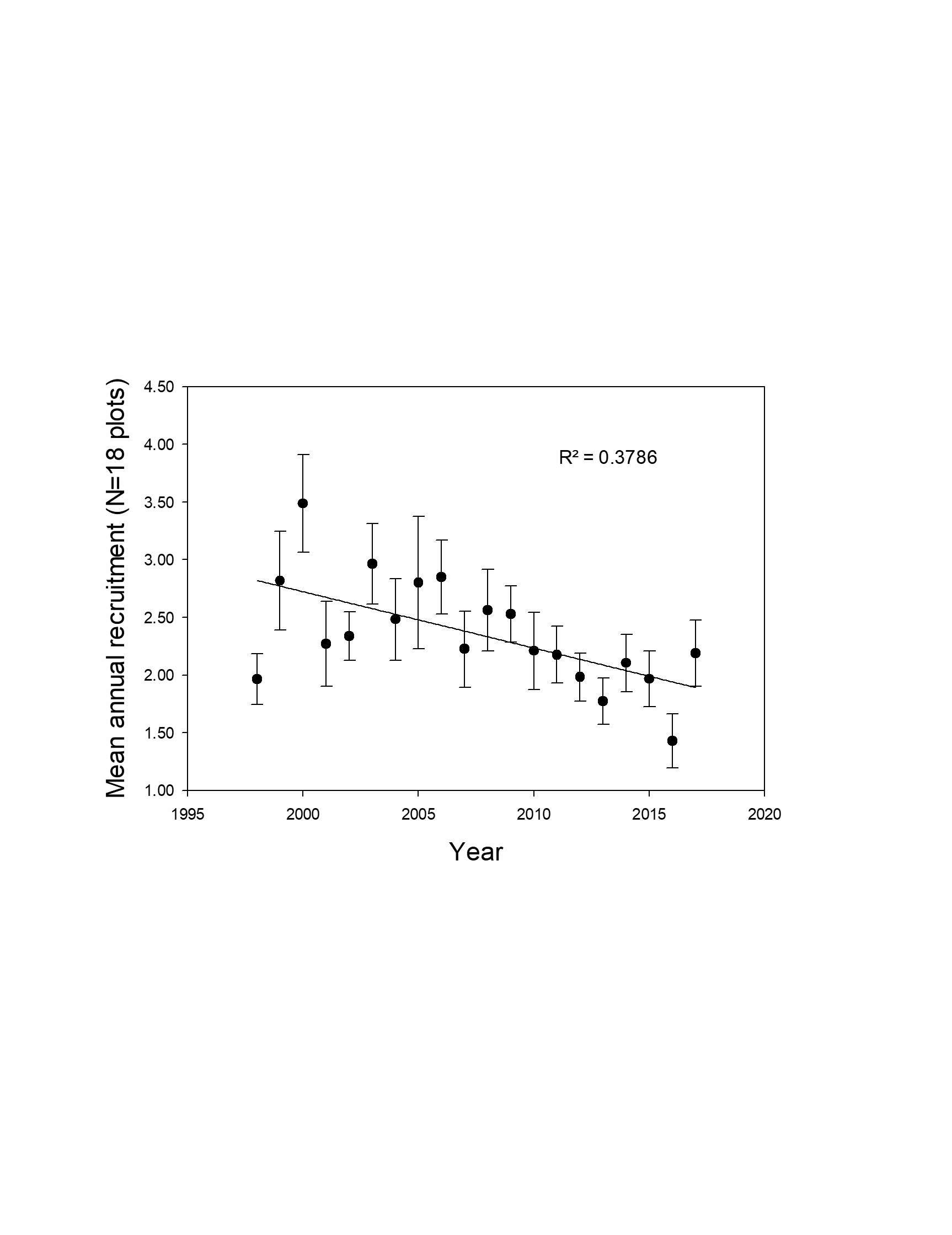

Supplement: S9 Fig — (TIF) [file pone.0224896.s016.tif]
